# RNA Binding Motif 5 (RBM5) in the CNS—Moving Beyond Cancer to Harness RNA Splicing to Mitigate the Consequences of Brain Injury

**DOI:** 10.3389/fnmol.2020.00126

**Published:** 2020-07-15

**Authors:** Travis C. Jackson, Patrick M. Kochanek

**Affiliations:** ^1^Morsani College of Medicine, USF Health Heart Institute, University of South Florida, Tampa, FL, United States; ^2^Morsani College of Medicine, Department of Molecular Pharmacology and Physiology, University of South Florida, Tampa, FL, United States; ^3^Safar Center for Resuscitation Research, Department of Critical Care Medicine, School of Medicine, University of Pittsburgh, Pittsburgh, PA, United States

**Keywords:** RBM5, brain, alternative splicing, neuronal death, gene expression, RNA-binding protein, traumatic brain injury, cerebral ischemia

## Abstract

Gene splicing modulates the potency of cell death effectors, alters neuropathological disease processes, influences neuronal recovery, but may also direct distinct mechanisms of secondary brain injury. Therapeutic targeting of RNA splicing is a promising avenue for next-generation CNS treatments. RNA-binding proteins (RBPs) regulate a variety of RNA species and are prime candidates in the hunt for druggable targets to manipulate and tailor gene-splicing responses in the brain. RBPs preferentially recognize unique consensus sequences in targeted mRNAs. Also, RBPs often contain multiple RNA-binding domains (RBDs)—each having a unique consensus sequence—suggesting the possibility that drugs could be developed to block individual functional domains, increasing the precision of RBP-targeting therapies. Empirical characterization of most RBPs is lacking and represents a major barrier to advance this emerging therapeutic area. There is a paucity of data on the role of RBPs in the brain including, identification of their unique mRNA targets, defining how CNS insults affect their levels and elucidating which RBPs (and individual domains within) to target to improve neurological outcomes. This review focuses on the state-of-the-art of the RBP tumor suppressor *RNA binding motif 5* (RBM5) in the CNS. We discuss its potent pro-death roles in cancer, which motivated our interest to study it in the brain. We review recent studies showing that RBM5 levels are increased after CNS trauma and that it promotes neuronal death *in vitro*. Finally, we conclude with recent reports on the first set of RBM5 regulated genes identified in the intact brain, and discuss how those findings provide new clues germane to its potential function(s) in the CNS, and pose new questions on its therapeutic utility to mitigate CNS injury.

## Introduction

The history of brain injury research is full of examples in which discoveries in cancer led to breakthroughs in novel neuroprotective strategies in the CNS. The symbiotic exchange of ideas between fields is based on the notion that temporarily inhibiting tumor suppressor genes might safely promote neuronal survival in the setting of an acute brain injury. RNA binding motif 5 (RBM5) is a ubiquitous nuclear splicing factor that regulates both exon definition (transcriptional variants) and total gene expression of select targets. In addition, it is a tumor suppressor gene. RBM5 mediated pro-death signaling pathways have been well characterized in cancer, and has resulted in increased recognition that its modulation may have therapeutic utility in the CNS. Independent of the expanding interest in RBM5 biology, increased awareness that disturbances in RNA splicing is a major contributing factor in the etiology of numerous human diseases, has stimulated the hunt for druggable targets that can modify gene splicing. Thus, RBM5′s unique trifecta of characteristics makes it an ideal target for additional study in the development of next-generation therapies for the CNS (i.e., its inhibition may promote neuronal survival, it can tailor gene splicing, and it can be modulated by small-molecules). In this review we will tether these ideas together—beginning with an overview of the basic splicing machinery. Next, we establish the importance in recognizing that damage to the spliceosome and gene-splicing is likely a major consequence of brain injury, and that its full impact on recovery remains nebulous pending much needed additional research. We highlight the evidence that RNA-binding proteins (RBPs) like RBM5 offer enormous potential to adjust splicing in the brain, as molecular gateways to the spliceosome. We consider the evidence that RBM5 is particularly attractive for that purpose because it promotes cell death in cancer cells and in neurons, and finally, discuss new evidence on its novel gene targets in the CNS, which has opened the door to new avenues of translational research.

## A Brief Review of The Spliceosome

Precursor messenger RNA (pre-mRNA) consists of alternating stretches of intronic and exonic (protein-coding) ribonucleotide sequences. Mature mRNA transcripts used for protein translation are produced by the excision of introns and stitching together of exons. In addition, unique cassette-exons can be either included (retained) or excluded (skipped), leading to a variety of mRNA transcripts that expands the functional repertoire of different proteins encoded by a single gene. In this focused review on RBM5 we will narrow discussions to encompass prototypical alternative cassette-exon events but acknowledge that the scope of RNA splicing is incredibly diverse. The reader is directed to in-depth resources on intron retention events (Braunschweig et al., 2014), long non-coding RNAs (Romero-Barrios et al., 2018), and circular RNAs (Wilusz, 2018; Li et al., 2019), among others, for a broader understanding of topics in splicing that are not essential here.

The processing of pre-mRNA transcripts in the spliceosome is regulated by coordinated rearrangements of specialized proteins. Moreover, their temporal incorporation, or exclusion, from the spliceosome advances stepwise in stages. Small nuclear ribonucleoproteins (snRNPs) are chief components of the basic splicing machinery and consist of a protein complexed with a small nuclear RNA (snRNA; Wassarman and Steitz, 1992). SnRNPs recognize key cis-acting RNA splicing elements found within pre-mRNAs and facilitate conformational changes in the transcript which are required to excise introns or selected cassette exons. The stages are defined as complex E’, E, A, B, and C. For simplicity we refer to them as steps (Berglund et al., 1998; Will and Lührmann, 2011; Matera and Wang, 2014). The prototypical steps involve binding of the U1 snRNP to the 5′ splicing donor site, and splicing factor 1 (SF1) binding to the branch point. This is followed by the U2 axillary protein (U2AF) binding to the polypyrimidine tract. The U2 snRNP is then recruited to the branch point and replaces SF1. This in turn exposes the 2′ hydroxyl group of the conserved adenosine nucleoside branch point to nucleophilic attack with the 5′ donor splice site. Finally, the U4/U5/U6 tri-snRNP complex is mobilized to induce several conformation changes in the transcript which brings into proximity the free hydroxyl group of the preceding exon with the 3’ acceptor splice site. The exons are ligated together and the intronic material is released as a “lariat loop.” This process is concurrent with gene transcription (Bentley, 2014). A host of additional splicing factors (SFs) and RNA-binding proteins (RBPs) transiently interact with the core spliceosomal machinery at various stages in the process to adjust exon-definition at select sites (Rappsilber et al., 2002; Jurica and Moore, 2003). RBM5 is a splicing modulator and its association with the spliceosome is increased at the complex A stage (Hartmuth et al., 2002).

## Disrupting The Spliceosome Is Cytotoxic: Evidence That Brain Injury Disturbs Core Components of The Splicing Machinery

Disruption of core components of the spliceosome robustly activates cell death. Tanackovic and Krämer (2005) used RNAi to inhibit either the splicing factor 3a (SF3A) or SF1 in HeLa cells. Disruption in the former produced necrosis while inhibition of the latter produced apoptosis (Tanackovic and Krämer, 2005). Consistent with the different cell death phenotypes, knockdown of SF3a broadly increased the number of intron-containing pre-mRNAs and resulted in a marked downregulation of total protein synthesis; thus, interfering with global pre-mRNA splicing decreased the available pool of translatable transcripts across the transcriptome which in turn resulted in a necrotic phenotype. In contrast, SF1 inhibition produced milder impairments on total protein expression and resulted in apoptosis, suggesting that dysregulation of a limited set of genes may have been responsible for cell death (Tanackovic and Krämer, 2005).

Few studies have examined the effect of brain injury on core components of the spliceosome. Several studies have found that SF1 is dysregulated after CNS injury. In a mouse model of experimental severe traumatic brain injury (TBI), SF1 protein levels were robustly decreased in the injured cortex/hippocampus between 4 h and 72 h post-injury (Jackson et al., 2015). Thus, TBI induced a sustained loss of SF1 in the brain. In contrast, SF3a levels showed little change post-injury and were relatively stable across the 4–72 h period. Covini et al. (1999) also found that SF1 mRNA levels were increased in resistant regions in the injured hippocampus 4 h post-ischemia in gerbils but conversely were depleted in dying CA1 neurons 4 days post-injury. It is unclear if SF1 downregulation in the brain is a causative or correlative factor in the setting of neuronal death. The aforementioned experiments by Tanackovic and Krämer (2005) in HeLa cells would suggest the former but this should be confirmed in primary neurons. Nevertheless, dysregulation of core splicing components (e.g., decreased SF1) might increase neuronal vulnerability to a subsequent insult, such as in the setting of a secondary brain injury after TBI. Supporting that notion, Sorrells et al. (2018) reported that mutant juvenile zebrafish engineered with an SF3b deficiency showed increased vulnerability to subsequent ionizing radiation (IR) injury, which increased caspase-3/p53-mediated apoptotic cell death in developing neurons. In that study, dysregulation of other core SFs (i.e., Txnl4a, Plrg1, Ccdc94, and Sfpq), which are involved at various stages of splicing (complexes B-C), also increased neuronal death after IR-injury in juvenile fish (Sorrells et al., 2018).

Thus, current (albeit limited) evidence suggests that some types of brain injury may temporally (or permanently) impair core components of the spliceosomal machinery. Next, we explore the potential impact of acute brain injury on alternative splicing of select genes and resultant translation of protein variants. It is unclear if changes in alternative splicing after injury are produced by underlying damage to the core spliceosomal complex (as discussed above), which might broadly disrupt biochemical equilibrium that normally favors certain variants, or if they result from disturbances in trans-acting factors (e.g., RBPs) that directly regulate exon definition (Lasko, 2003). Both may be involved. Regardless of the mechanism(s) that drives splicing changes after an insult, we speculate that sustained maladaptive alternative splicing might lead to, or be a unique manifestation of, a secondary brain injury that may represent a key cell death effector and therapeutic target to restore normal brain function and improve cognitive health (Xiong et al., 2015; Li et al., 2016).

## CNS Injury Alters Alternative Splicing: Evidence from Genome-Wide and Focused Gene-Level Studies

Acute brain injury is well-known to alter gene splicing and has been confirmed using either big data approaches (e.g., global transcriptomic screening) or analysis of targeted mRNAs (e.g., RT-PCR to quantify specific genes and their transcripts of interest). Recent whole-genome microarray studies identified 564 significant splicing events in the cortex at 48 h after a severe TBI in male and female wild-type CRE+ littermates of RBM5^tm1Ozg^ mice (Jackson et al., 2020). The majority of these events (341) were classified as cassette-exons. Among the genes identified and that have a known role in modifying outcomes after a brain injury were: caspase-8, matrix metallopeptidase 12 (MMP-12), serpina3g/Spi2A, TNF receptor superfamily member 1A (TNFR1), glial fibrillary acidic protein (GFAP), and tissue inhibitor of metalloproteinase 1 (TIMP-1). Notable exons that were robustly affected by TBI included: (a) exon 4 in the Serpina3g transcript which increased 43.32-fold after a TBI; and (b) exon 1 in GFAP which decreased 21.9-fold (Jackson et al., 2020). The effect (beneficial or detrimental) that these changes have on CNS recovery is unknown. Similarly, in a *Drosophila* (fruit fly) model of mild TBI, 578 differentially expressed splicing events were detected in the brains of male and female flies 24 h post-injury and additional sex-specific events were also detected (Sen et al., 2017). Ischemic brain injury also affects splicing. RNAseq studies on whole blood isolated from humans diagnosed with stroke identified 412 differentially expressed splicing events vs. controls. Remarkably, distinct mechanisms and subtypes of stroke produced unique patterns of differential gene-splicing (e.g., intracranial hemorrhage vs. embolic vs. large vessel or small vessel lacunar ischemic stroke; Dykstra-Aiello et al., 2015). Chronic brain diseases may cause even greater impairment of spliceosomal homeostasis. RNAseq studies on human brain tissue in patients with Alzheimer’s disease (AD) vs. controls found 422 transcripts in the temporal lobe and 927 in the frontal lobe that were *only expressed* in diseased individuals (Twine et al., 2011). Similarly, recent studies found that Tau tangles trap numerous core components of the spliceosome machinery leading to widespread splicing errors, and the burden of Tau pathology in human AD brains correlated with the extent of spliceosomal disruption (Hsieh et al., 2019). Given that experimental TBI in mice produced sustained and malignant spread of Tau pathology in the injured brain, splicing aberrations may progressively worsen after a CNS insult (Edwards et al., 2020). Gradual worsening of spliceosomal homeostasis after acute brain injury, might suggest a broad therapeutic time window to intervene using splicing directed therapies.

Studies on individual gene targets have provided a more focused characterization of notable splicing events induced by CNS injury. For instance, alternative splicing produces two transcripts of the microtubule protein Tau (3R and 4R); the ratio of 3R/4R mRNA is increased in the spinal cord 14 days after a peripheral nerve transection in rats and maintained for at least 42 days (Chambers and Muma, 1997). Splice variants of amyloid precursor protein (APP) including APP751 and APP770 contain the Kunitz-type protease inhibitor (KPI) domain which blocks enzymatic degradation by serine proteases and may contribute to the pathological deposition of Aβ in the setting of AD; rats given a fluid-percussion TBI have increased APP751/770 levels 24 h post-injury but decreased cortical levels of a variant that lacks the KPI domain (APP695) 1 h post-injury (Masumura et al., 2000). Presumably, therapeutically interfering with APP splicing to favor APP695 would be beneficial in TBI. The gene for the glutamatergic ion channel α-amino-3-hydroxy-5-methyl-4-isoxazolepropionic acid (AMPA) receptor, encodes multiple transcripts including variants flip vs. flop; the ratio of flip/flop mRNA levels is increased in the spinal cord of patients with neurodegenerative amyotrophic lateral sclerosis (Tomiyama et al., 2002). The enzyme tissue transglutaminase (tTG) is involved in apoptosis (Melino and Piacentini, 1998); traumatic spinal cord injury (SCI) in rats induces the expression of a second short-variant (tTGs) 8 h post-injury (Festoff et al., 2002). Prosaposin (SGP1) is a secreted protective factor that promotes recovery of injured myelinating glia/neurons and some transcripts have, whereas others lack exon 8. The ratio of SGP1 transcripts with/without exon 8 is 85:15 in the normal rat CNS but is reversed to 5:95 after brain ischemia (Hiraiwa et al., 2003). There are many other examples in which the ratio of splice variants for genes is altered after CNS or PNS damage including: neuregulin-1 (Kerber et al., 2003), glutamate transporter 1 (Yi et al., 2005), growth arrest-specific gene 7 (Chang et al., 2005), Tenascin-C (Dobbertin et al., 2010), fibronectin (Khan et al., 2012), and Bcl-x (Xiao et al., 2012). Unfortunately, for most of these genes the manner in which alternative splicing effects neuronal damage or cognitive outcomes post-injury is still unknown. Nevertheless, based on a handful of gene-splicing studies in which a CNS functional change has been established—it is clear that it can have profound effects. For instance, variants of apolipoprotein E receptor 2 (ApoER2) lacking exon 16 robustly modify hippocampal spine density, glutamate receptor levels, and increase long-term potentiation in mice (Wasser et al., 2014). Given the myriad of acute and/or chronic splicing events that occur after CNS injury, the consequences of altered splicing on brain function may be substantial. More research is needed to determine if correcting spliceosomal imbalances is feasible and if it holds promise as a new treatment. One concept to correct aberrant splicing is to target RBPs and is discussed next. Other proposed approaches to modulate RNA splicing are outside the scope of this review.

## RNA-Binding Proteins as Therapeutic Targets to Modulate Transcript Definitions: Progress on RBM5 Inhibitors

A recent surge of disease-focused reviews have highlighted the emerging clinical interest for therapeutically targeting RBPs in the setting of: neurodegenerative diseases (Hofmann et al., 2019; Nussbacher et al., 2019), pain disorders (de la Pena and Campbell, 2018), cancer (Pereira et al., 2017), immunity (Turner and Díaz-Muñoz, 2018), diabetes (Nutter and Kuyumcu-Martinez, 2018), muscle wasting (Van Pelt et al., 2019), reproductive pathologies (Khalaj et al., 2017), cardiovascular disease (de Bruin et al., 2017), renal injury (Ignarski et al., 2019) and hepatic illness (Lee et al., 2020). Increased awareness of RBPs as targets has accelerated efforts to develop therapies that disrupt and/or modulate their activity. Small-molecule pharmacological inhibitors are particularly appealing due to their inherent design flexibility (stereochemistry). This may allow for the design of molecules that selectively interfere with specific binding regions of an RBP (i.e., a single functional domain), which could limit off-target effects caused by complete inactivation of the entire protein (e.g., such as with RNAi).

Small-molecule approaches to modify mRNA-protein interactions are in development for numerous RBPs and include novel drugs to target: human antigen R (Wu et al., 2015), Musashi RNA binding protein 2 (Minuesa et al., 2019), human immunodeficiency virus (HIV) Rev protein (Zapp et al., 1993), and spinal muscular atrophy (SMA) 2 splicing (Wang et al., 2018). Pharma is scaling up investment in this emerging therapeutic space. A prime example is Anima Biotech, which is developing transcript-modifying approaches to treat human diseases. If successful, their Translation Control Therapeutic Platform might greatly expand the percentage of the druggable proteome that can be targeted with small-molecules (i.e., historically, enzymes or ligand receptors have been the main focus of drug development; Verdine and Walensky, 2007).

**Figure 1:**
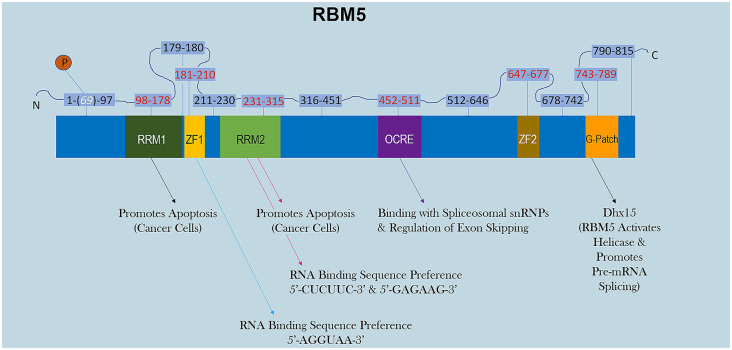
RBM5 protein structure. The illustration shows the major canonical functional domains in the human RBM5 protein, and regions which may be amenable to small-molecule inhibitors. The amino acid distance spanning each functional domain is indicated in red text. Serine 69 is phosphorylated (white text). Arrows indicate the known interactions of each domain’s binding partner(s) and/or function. All studies relevant to the depictions in the diagram are cited in the review. RNA Recognition Domain (RRM), Zinc-Finger Domain (ZF), Octamer Repeat (OCRE) Domain, Glycine-Rich Region (G-Patch) Domain, DEAH-Box Helicase 15 (Dhx15).

RBM5 has multiple RNA-binding domains that include two RNA Recognition Motif (RRM), and two zing-finger (ZF) domains (Figure 1). The RRM and ZF domains are thought to modulate distinct sets of mRNAs (Farina et al., 2011; Song et al., 2012). Additional domains in RBM5 (e.g., g-patch) help to coordinate its activity during RNA splicing but the specificity of those effects (i.e., if limited to certain mRNAs or rather functions in a promiscuous manner) remain to be determined (Niu et al., 2012; Mourao et al., 2016). The functional roles of each RBD are only just beginning to come to light. Recent reports in cancer cells found that the RRM domains in RBM5 (but not the two ZF domains) were necessary for its pro-death functions (Zhang et al., 2014). Whether or not the RRM domains in RBM5 regulates cell death mRNAs in other cell types including neurons remains unclear. Also, the cell signaling functions of the ZF domains remain to be determined. Germane to potential RBM5 inhibitors, there are currently no drugs to block the pro-death RRMs. In contrast, the compound anthraquinone-2-sulfonic acid (AQ2S) reportedly inhibits (competitively) the RAN-BP2 type ZF in the RBM5 domain with a dissociation constant (KD) of ~82 μM to interfere with binding of small-RNAs (Farina et al., 2011). However, those experiments were done in a cell-free system and the potential (and/or potency) of AQ2S to block the RBM5-ZF domain in living cells has yet to be determined. Intriguingly, we reported that AQ2S inhibited cell death in injured primary rat cortical neurons *in vitro* but later discovered that the mechanism involved direct inhibition of multiple pro-death caspases (Jackson et al., 2013). Whether AQ2S modifies facets of RBM5 functions in neurons linked to the ZF domain, such as by altering splicing of mRNA targets, remains to be elucidated. Thus, pharmacological approaches to inhibit RBM5 is a promising area of research but more work is needed to identify/expand the repertoire of small-molecule “RNA mimetics” for individual domains, which will also help to investigate each domain and its unique role in either promoting or reversing neuropathology after a brain injury.

Optimizing small-molecules such that they effectively cross the blood-brain barrier (BBB) is a major challenge in CNS therapeutics (Pajouhesh and Lenz, 2005). In practice it would be ideal if lead compound optimization processes were able to focus primarily on facets of the molecular objective at the site of action (e.g., defining the most potent stereochemistry that inhibits a kinase domain, blocks a receptor, or interferes with RNA-protein interactions modulated by RBPs) rather than simultaneously having to consider moieties that either promote or hinder brain disposition. Fortunately, CNS-drug delivery systems are advancing at a rapid pace and may eventually eliminate the need to prioritize chemical structures based on their inherent ability to penetrate the BBB. Promising technologies for enhancing the delivery of drugs and other small-molecules to the brain includes: cyclic peptides (Yamaguchi et al., 2020), focused ultrasound with gaseous microbubbles (Chen et al., 2019), nanoparticle/receptor transport agents (Saraiva et al., 2016), and extracellular vesicles (Wiklander et al., 2019). These drug-delivery systems have broad utility and implications for neurotherapeutics that extend well beyond the topic of this review. Nevertheless, their brief mention here is pertinent because novel RBP inhibitors may require more efficient ways to target the brain as is the case with many other promising or well-known neuroprotective drugs currently in use (Sanchez-Lopez et al., 2018).

## RBM5 Is A Potent Pro-Death Effector in Cancer Cells: A Window into Its Potential as A Therapeutic Target After CNS Injury

**Figure 2:**
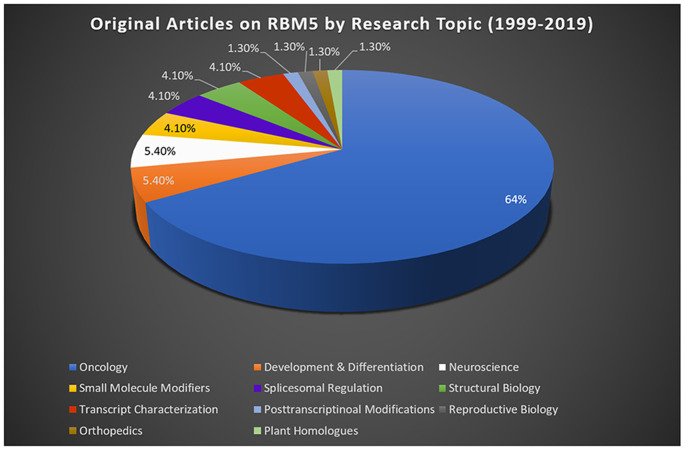
RBM5 literature and state-of-the-art. The search term “RBM5” was imputed into *PubMed* and articles examined across a 20-year epoch, spanning from the discovery of RBM5 in 1999 up until 2019. A total of 95 items were identified at the time of the search. All articles were screened for potential exclusions (22 total). The reasons for exclusions included: reviews (eight articles), lack of data relevant to RBM5 after additional scrutiny of study details (10 articles), abstracts only (two articles), not in English (one article), and editorials germane to a published original work that was already included in the analysis (one editorial). A total of 73 articles remained: oncology (47 articles), development and differentiation (four articles), neuroscience (four articles), small-molecule modifiers (three articles), spliceosomal regulation (three articles), structural biology (three articles), transcript characterization (three articles), orthopedics (one article), post-transcriptional modifications (one article), reproductive biology (one article), and plant homologs (one article). Article categorization was decided by the topic that best fits the experiments performed and goals of the study but is our interpretation and does not necessarily reflect the author’s views.

Most studies reporting on RBM5 mediated mechanisms that regulate cell death were done in cancer. Sixty-four percent of publications on RBM5 indexed in *PubMed* between 1999 (the year it was discovered) and 2019 were in the field of oncology (Figure 2). The focus of cancer research on RBM5 is the result of its location in the 3p21.3 gene cluster. The 3p21.3 loci in chromosome 3 includes a ~370 kb region which encodes 19 potential tumor suppressor genes that are frequently inhibited in tumors (Lerman and Minna, 2000). Loss of heterozygosity in the 3p arm is among the earliest pathological events detected in many cancers (Euhus et al., 1999; Martinez et al., 2001). Over the past two decades, extensive research on those 19 genes (including RBM5) sought to elucidate their cell signaling functions and to identify which among them exerts the greatest influence to drive tumorigenesis and metastasis when downregulated. The history of investigations on RBM5 in cancer is relevant here because the large body of foundational work in that field is important to understanding its possible neurotoxicity in the brain.

**Figure 3:**
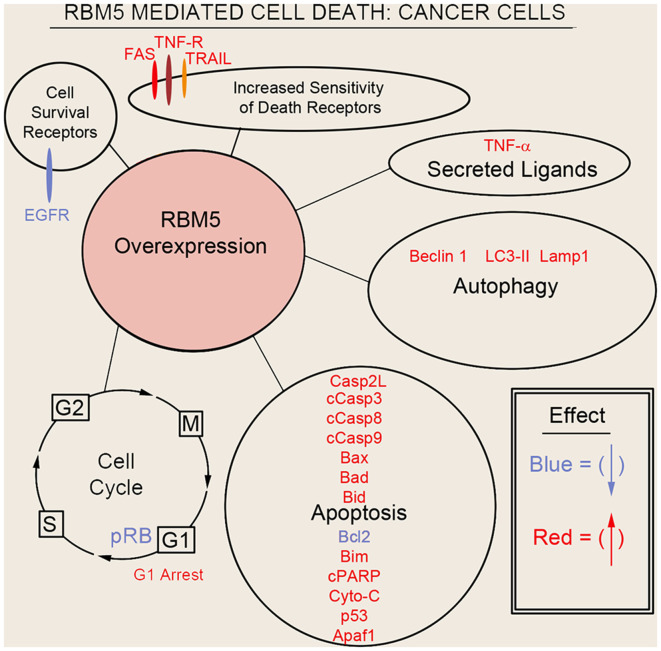
Mechanisms of RBM5-mediated cell death in cancer. The major signaling mechanisms mediating RBM5 pro-death activity are illustrated. Different processes are separated by circles/ovals. Most of the evidence comes from studies in which RBM5 was overexpressed. Targets in red indicate that their levels were increased with RBM5 overexpression. Targets in blue indicate their levels were decreased with RBM5 overexpression. All studies supporting the depictions in the diagram are cited in the main text. Apoptosis antigen 1 (APO-1/FAS), Tumor necrosis factor receptor (TNF-R), TNF-related apoptosis-inducing ligand (TRAIL), Tumor necrosis factor-α (TNF-α), Microtubule-associated protein 1A/1B-light chain 3 II (LC3-II), Lysosomal-associated membrane protein 1 (LAMP1), Caspase-2-long (Casp2L), Cleaved caspase-3 (cCasp3), Cleaved caspase-8 (cCasp8), Cleaved caspase-9 (cCasp9), BCL2 Associated X (BAX), Bcl2 antagonist of cell death (BAD), BH3 Interacting Domain Death Agonist (BID), B-cell CLL/lymphoma 2 (BCL2), Bcl-2-like 11 (Bcl-2L11/BIM), Cleaved poly (ADP-ribose) polymerase (cPARP), Cytochrome c (Cyto-C), cellular tumor antigen p53 (p53), Apoptotic protease activating factor 1 (Apaf1), phosphorylated retinoblastoma protein (pRB), G_2_ Interphase (G2), Mitosis and cytokinesis phase (M), G_1_ phase (G1), Synthesis phase (S), epidermal growth factor receptor (EGFR).

Sutherland et al. (2010) pioneered the potent tumor suppressor effects of RBM5 in multiple cancer cell types. Two decades of molecular studies in which RBM5 was either experimentally knocked down or overexpressed has reproducibly shown that it promotes cell survival or cell death, respectively. A host of pro-death signaling mechanisms regulated by RBM5 has been elucidated (Figure 3). For clarity we divide these mechanisms into five major categories: (1) increased activation of apoptotic processes (Oh et al., 2006; Fushimi et al., 2008; Kobayashi et al., 2011; Li et al., 2012; Shao et al., 2012, 2013; Zhao et al., 2012; Su et al., 2016; Jiang et al., 2017); (2) increased activation of autophagic processes (Su et al., 2016); (3) increased sensitization and stimulation of extracellular death receptors and extrinsic apoptotic pathways (Rintala-Maki and Sutherland, 2004; Shao et al., 2013; Jiang et al., 2017); (4) downregulation of pro-survival growth factors (Su et al., 2014); and (5) increased cell cycle arrest (Oh et al., 2006; Xiao et al., 2014; Lv et al., 2016). The host of mechanisms by which RBM5 promotes pro-death pathways limits tumorigenesis *in vivo*. Several investigators reported that increasing RBM5 levels *via* injection of vector plasmids in immunocompromised nude mice inoculated with A549 or BEAS-2B cancer cells inhibited tumor growth (Oh et al., 2006; Shao et al., 2012, 2013; Lv et al., 2016).

Recent *in vivo* studies using novel RBM5 transgenic mice have shed additional light on its tumorigenic role *in vivo*. Hemizygous total body RBM5 gene deleters administered the potent cigarette-smoking carcinogen, 4-(methylnitrosamino)-1-(3-pyridyl)-1-butanone (NNK), had increased tumor burden by 48 weeks, but not earlier (Jamsai et al., 2017). In other studies, homozygous mice with an inactivating point mutation in the 2nd RRM domain of RBM5, rendering it non-functional, were healthy and showed no overt signs of lung cancer by 9 months of age (O’Bryan et al., 2013). The absence of tumorigenicity in homozygous mice with an inactive 2nd RRM domain further supports the notion that developing small-molecule agents to selectively block individual functional domains within RBM5 may provide a therapeutic approach to targeting RBPs that poses a lower risk for developing complications like cancer (e.g., such as in patients treated for brain injury).

## Empirical Evidence That RBM5 Promotes Neurotoxicity in The CNS: Where Do We Stand?

As of 2019 about 5.4% of the RBM5 literature indexed in *PubMed* concerns the field of neuroscience, including acute brain injury research, neurodegeneration, neurodevelopment, and neurobiology (Figure 2). Furthermore, the first reports on RBM5 in the CNS were published in 2015. This included an observational study on the endogenous changes in RBM5 protein levels after experimental TBI in mice (Jackson et al., 2015) and an observational study by Zhang et al. (2015) on its endogenous levels after SCI in rats. The conclusion independently reached by both groups was that RBM5 protein levels are increased in neural tissue after injury. However, the magnitude of the induction and the duration of the increase were markedly greater in SCI vs. TBI; indeed, both RBM5 mRNA and protein levels rapidly increased 6 h post-injury in the spinal cord and remained elevated for 7 days (Zhang et al., 2015). After TBI, RBM5 staining in the hippocampus was markedly increased at 48 h. After TBI, the RBM10 paralogue showed even greater induction in the hippocampus and in cortex vs. RBM5 at 48 h (Jackson et al., 2015). Finally, prior gene expression studies had shown that the brain is among the organs with the highest expression of RBM5 mRNA. Jackson et al. (2015) confirmed that protein levels are concentrated in neurons and are expressed at lower levels in glia (e.g., astrocytes).

Both of the aforementioned studies supplemented observational studies with *in vitro* cell culture experiments—to directly test if RBM5 inhibition decreased neuronal death and thus infer whether or not increased levels after CNS injury might exacerbate cell death. Zhang et al. (2015) used hydrogen peroxide (H_2_O_2_) to injure rat neuronal PC12 cells, which induces a necrotic-like form of cell death. RBM5 levels in H_2_O_2_ treated PC12 cells increased 6–12 h post-injury. Blocking its induction with RNAi before H_2_O_2_ injury decreased the levels of p53 and inhibited caspase-3 activation (Zhang et al., 2015). This finding is consistent with the notion that increased RBM5 levels after a CNS injury could be harmful to the CNS. Jackson et al. (2015) used a similar *in vitro* model to test the hypothesis. Retinoic-acid differentiated human neuronal SHSY5Y cells were treated with staurosporine (STS) to induce apoptotic cell death. Endogenous RBM5 protein levels did not increase in injured SHSY5Ys at 8 h after STS (Jackson et al., 2015). Nevertheless, RBM5 RNAi decreased STS-induced PARP cleavage, decreased caspase-9 cleavage, and reduced the levels of a non-specific ~70 kDa caspase-cleavage product (Jackson et al., 2015). Taken together the evidence suggest that: (a) increased RBM5 after a CNS injury may be detrimental, and (b) blocking RBM5 may represent a novel neuroprotective strategy.

Subsequent studies evaluated RBM5 toxicity in mixed primary rat cortical neuron/astrocyte cultures. These studies employed a model of mechanical stretch-injury which replicates components of a brain trauma *in vitro*, and links cell culture findings to prior work in TBI. Endogenous RBM5 was not increased in primary cortical neurons 24 h after a single or multiple mechanical stretch-injury insults (Jackson et al., 2018). This stretch-injury model produces neuropathological changes more consistent with a mild TBI *in vivo*. Thus, the level of insult severity may be an important modulator of increased endogenous RBM5 expression. However, more studies are needed to determine if different types of insults, at different severity levels, modify the threshold for RBM5 induction in neurons/brain. RBM5 overexpression with a lentivirus exacerbated cell death after a stretch-injury in primary neurons and was confirmed both by increased levels of α-spectrin breakdown products (i.e., a molecular readout of calpain activation) and by increased levels of lactate dehydrogenase in the media 24 h post-injury (Jackson et al., 2018). Thus, consistent with findings in immortalized neuronal-like cells, RBM5 enhanced neurotoxicity in primary neurons. Studies by our group are underway to measure histological and behavioral outcomes after experimental TBI in novel conditional RBM5 KO mice, which should shed new light on the neuroprotective potential of blocking RBM5 after a brain injury *in vivo*.

## RBM5-Mediated Splicing Targets: Cell Death Effectors in Cancer Vs. Novel Gene Targets in The CNS

**Figure 4:**
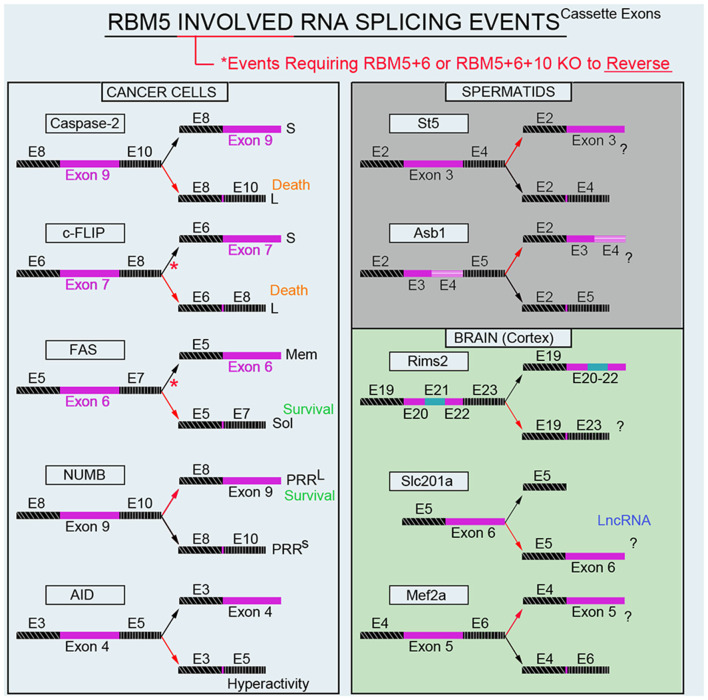
RBM5-regulated gene-splicing targets in cancer lines, testes, and in cortical brain tissue. Spliced cassette exon(s) are indicated in maroon. Red arrows indicate the direction of splicing that RBM5 favors. Red asterisks indicate that RBM5 and one or more of its paralogues (RBM6 or RBM10) had to be simultaneously inhibited to shift the gene-splicing event in the opposite direction. All studies relevant to the depictions in the diagram are cited in the main text. Cellular FLICE-like inhibitory protein (c-FLIP), apoptosis antigen 1 (APO-1/FAS), NUMB endocytic adaptor protein (NUMB), Activation-induced cytidine deaminase (AID), Suppression of tumorigenicity 5 (St5), Ankyrin repeat and SOCS box containing 1 (Asb1), Regulating synaptic membrane exocytosis 2 (Rims2), phosphate (Pi) transporter slc201a (Slc201a), Myocyte enhancer factor 2A (Mef2a).

Modulation of RNA splicing is a key mechanism by which RBM5 inhibits the proliferation and survival of cancer cells. Indeed, RBM5 regulates the inclusion of cassette-exons in caspase-2 (Fushimi et al., 2008), cellular flice-like inhibitory protein (c-FLIP; Bonnal et al., 2008), and NUMB (Bechara et al., 2013), and in a manner that favors the translation of protein variants which have increased toxicity (Figure 4). In contrast, RBM5 also alters the cassette-exon definition of apoptosis antigen 1 (FAS receptor) in a manner that favors pro-survival transcripts (Bonnal et al., 2008). RBM5 also alters the splicing of activation-induced cytidine deaminase (AID) which modulates DNA mutagenesis (Jin et al., 2012). Most of the data on these splicing events come from *in vitro* studies in transformed cell lines. Thus, the extent that RBM5-mediated changes in the splicing of these targets promotes cell death *in vivo* remains to be determined. Also, for many of these genes the alternatively spliced transcripts have been detected in non-cancerous organs; i.e., both caspase-2 short and long variants are found in the rat brain (Jin et al., 2002). The extent that RBM5 modifies their exon-definition in normal (non-transformed) vs. cancerous tissue remains unclear.

The lack of studies on the role of RBM5 in healthy tissues has led to a paucity of data on its targets in non-transformed cells, and whether or not they differ or overlap from those reported in cancer. We are addressing that knowledge gap in the CNS. Cell culture studies on human neuronal-like SHSY5Ys revealed that RBM5 knockdown increased the levels of the pro-survival (short) variant of c-FLIP (Jackson et al., 2015). Also, splicing of the gene amyloid precursor-like protein 2 (APLP2) appeared changed, based on the observation that banding patterns of APLP2 proteins detected on SDS-PAGE differed in RBM5 knockdown vs. controls cells. In contrast, APLP2 banding on SDS-PAGE did not differ in RBM10 knockdown vs. control SHSY5Ys.

Microarray analysis of immature primary rat cortical neurons transduced with a lentivirus to either knockdown or overexpress RBM5 resulted in a limited number of gene changes (Jackson et al., 2017). The most robust changes were seen in RBM5 knockdown neurons and included increased mRNA levels of Sec23a, a gene involved in vesicular trafficking, and also increased levels of the small GTPase Rab4a which regulates endocytosis of receptors involved in neurotransmission (Jackson et al., 2017). At the protein level, only Rab4a was increased in RBM5 knockdown neurons (Jackson et al., 2017). Furthermore, increased Rab4a correlated with decreased plasma membrane levels of the oligomeric form of the serotonin transporter (SERT), as well as increased cytoplasmic levels of endocytosed monomeric SERT. This suggested that RBM5 might preferentially regulate genes involved in neurotransmission in the CNS rather than pro-death genes as reported in cancer. Surprisingly however, RBM5 overexpression did not alter gene expression in immature cortical neurons nor did it result in decreased Sec23a/Rab4a mRNA levels below baseline. Lentiviral vector manipulations raised RBM5 protein levels ~2-fold above control levels, which may have achieved a sub-threshold increase needed to alter gene expression. More studies are needed to explore that possibility and to determine if gene dosing influences RBM5 toxicity. Several additional limitations merit mention. First, the absence of an injured group may have precluded the detection of RBM5-mediated changes to pro-death genes in immature neurons. Second, because cell culture procedures rely on constantly-evolving methodologies, the choice of experimental parameters may influence study outcomes. For instance, we reported in primary rat cortical neurons that the new cell culture medium BrainPhys/SM1 robustly increases the background expression of critical CNS proteins associated with normal brain function *in vivo* vs. gold-standard Neurobasal/B27 (Jackson et al., 2018). RBM5 investigations in immature cortical neurons were performed using Neurobasal/B27 and could have influenced the targets identified in RBM5 knockdown neurons. Similarly, the effect of culture medium on prior studies of RBM5 in cancer cells could impact the observed gene-splicing targets.

Recently we conducted global gene expression analysis and investigated splicing changes in cortical tissue from brain-specific RBM5 KO mice (Jackson et al., 2020). To clarify if a brain injury is needed to boost the detection of RBM5-regulated events linked to cell death genes (e.g., pro-death caspases) we included shams and mice given a severe controlled cortical impact (CCI) TBI. A variety of pro-death genes were upregulated after a TBI. This included increased levels of caspases and FAS (both which were previously reported to be regulated by RBM5 in cancer cells). However, neither the total expression levels nor gene-splicing of these targets was altered by RBM5 KO after CCI. Instead, a shortlist of novel differentially expressed gene targets were identified in the KO cortex and included *casein kinase* 2 s*ubunit alpha’ interacting protein* (Csnka2ip), Gm756, Serpina3n, and GFAP. A total of 22 novel alternative splicing events were detected across 18 genes. Several of these cassette-exon splicing events were further confirmed by quantitative Nanostring. This included in KOs: (a) increased inclusion of a tri-exon block spanning exons 20–22 in *regulating synaptic membrane exocytosis 2* (Rims2), (b) increased exclusion of exon 6 in a long non-coding RNA transcript of the phosphate handling (Pi) transporter Slc201a gene, and (c) increased exclusion of exon 5 in *myocyte enhancer factor 2A* (Mef2a; Figure 4). Nanostring also confirmed increased inclusion of exon 3 in N-myc downstream-regulated gene 2 protein (Ndrg2) in the KO cortex; however, the difference was modest and only detected in shams. *In situ* hybridization (ISH) against Rims2 further confirmed increased Rims2 splicing in the KO cortex and revealed increased transcripts in the hippocampus as well. Finally, total Rims2 staining increased ~4-fold in the KO cerebellum. Thus, Rims2 was among the genes most effected by RBM5 KO in the intact brain. Rab4a mRNA was unaffected in the KO vs. WT cortex, contrary to findings on its levels in cultured rat cortical neurons after lentiviral-induced RBM5 knockdown (Jackson et al., 2017). Experimental differences between *in vivo* vs. *in vitro* studies might explain the discrepancy and including, the species used, and the developmental age, among other factors. However, an alias for Rims2 is *Rab3-interacting protein*. Therefore, while Rab4 was not altered in the RBM5 KO brain *in vivo—*a Rab signaling pathway effector (Rims2) was among the genes most affected by RBM5 deletion, and hints that the similarities in theme across studies may not be coincidental.

The brain and testes are among the organs with the highest baseline expression levels of RBM5 in the body (O’Bryan et al., 2013). While we are focused on elucidating its gene targets in the CNS, others have studied its targets in the testes. Mutant mice with an impaired 2nd RRM domain in the RBM5 protein had altered splicing of *suppression of tumorigenicity 5* (St5) and *ankyrin repeat and SOCS box protein 1* (Asb1) in the adult testes (O’Bryan et al., 2013; Figure 4). While these genes were distinct from those identified in our screen of the brain, St5 is a potent regulator of Rab9A (Yoshimura et al., 2010), Rab9B (Yoshimura et al., 2010), and Rab13 (Ioannou et al., 2015). Thus, while RBM5 appears to target genes in a highly tissue-specific manner, the pattern that reemerges is that it regulates Rab signaling pathways. More research is needed to understand the extent that RBM5 may regulate exocytosis and/or endocytosis given that much of its effects on those processes may be mediated by indirect mechanisms and thus not well captured by global gene expression studies of the transcriptome.

Recent data on RBM5 in the CNS has thus revealed a number of exciting findings relevant to novel gene targets that merit future investigation. Cumulative data from RNAi studies to elucidate genes and/or their splicing regulated by RBM5 in human neuronal-like cells, immature primary rat cortical neurons, and intact brain tissue in adult mice, increasingly suggest that RBM5 plays a distinct role in the CNS vs. its role in cancer. On the other hand, *in vitro* CNS injury studies support the notion that RBM5 increases neuronal vulnerability to an insult, much like its effect on cancer cells to augment cell death after exposure to chemotherapeutic agents (Loiselle et al., 2016). The mechanisms mediating increased vulnerability between neurons vs. cancer cells may differ based on differences in the genes that are affected by RBM5 inhibition. Future studies are needed to establish that RBM5 KO is neuroprotective *in vivo*. Finally, because Rims2 regulates neurotransmitter release at the synapse, and RBM5 alters its splicing in the cortex and hippocampus, it is possible that KOs have altered phenotypes germane to behavioral recovery after an insult independent of potential changes in histological neuroprotection (Kaeser et al., 2012; Jackson et al., 2020). Research is underway to address that possibility.

## Discussion

RBM5 brain research is in its infancy. Fundamental questions on its role in neurobiology and on the mechanisms of its regulation remain to be answered (e.g., what is the effect of posttranslational phosphorylation on its activity; Shu et al., 2007). RBPs are an emerging new class of therapeutic targets and RBM5 is a promising candidate among them to test if modifying its activity adjusts RNA splicing in a manner that promotes post-insult recovery in the CNS. Initial *in vitro* findings indicate that RBM5 inhibition decreases cell death/injury after an insult in neurons. The functional domains (e.g., RRMs or ZFs) responsible for promoting increased vulnerability to injury remain to be elucidated. Also, the RBD that promotes Rims2 splicing has yet to be identified and may be different from the RBD that promotes cell death. If true, it could open the door to a suite of theoretical drugs to tailor RBM5 activity for different therapeutic purposes.

**Figure 5:**
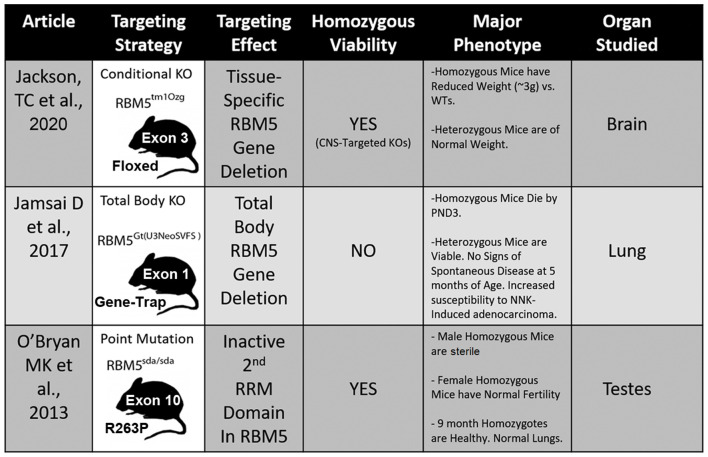
Transgenic models to study RBM5. The table illustrates the three available genetic mouse models to investigate RBM5 signaling. The targeting strategy, overall strain viability, primary tissue investigated in each animal, and major phenotypes are indicated for each strain.

Three different transgenic models have been created to investigate RBM5 biology *in vivo* and each has unique advantages and disadvantages to elucidate its signaling functions (Figure 5; O’Bryan et al., 2013; Jamsai et al., 2017; Jackson et al., 2020). To date, all three transgenic mice incorporate a mutational change that results in the inactivation of RBM5 (either partially or completely). Additional animal models are needed to study the effect of RBM5 overexpression in brain injury. Also, RBM5 overexpressing mice could help to clarify the potential for gene-dosing effects of RBM5 on cell death pathways after injury, if they occur.

RBM5 KO mice also need to be studied in pre-clinical models of CNS injury besides trauma (e.g., stroke, cardiac arrest, and neurodegenerative diseases). Unique changes in the global transcriptome induced by different types of insults may modify the gene targets regulated by RBM5, thus effecting its therapeutic potential depending on the insult. Moreover, global gene expression changes need to be measured across the acute and chronic post-injury periods, and in young vs. adult mice, given that age robustly influences gene expression changes after a CNS insult (Cho et al., 2016). The new therapeutic opportunities afforded by investigating the modulation of RBP SFs promises to include many unexpected findings—and it is time to move beyond cancer to harness RNA splicing to develop new potential strategies to mitigate the consequences of CNS injury.

## Author Contributions

TJ and PK contributed equally to this work.

## Conflict of Interest

Dr. TJ and Dr. PK are inventors on a USPTO patent (No. 9,610,266) titled Small molecule inhibitors of RNA binding motif (RBM) proteins for the treatment of acute cellular injury.
